# Contribution for the Derivation of a Soil Screening Value (SSV) for Uranium, Using a Natural Reference Soil

**DOI:** 10.1371/journal.pone.0108041

**Published:** 2014-10-29

**Authors:** Ana Luisa Caetano, Catarina R. Marques, Ana Gavina, Fernando Carvalho, Fernando Gonçalves, Eduardo Ferreira da Silva, Ruth Pereira

**Affiliations:** 1 Department of Biology, University of Aveiro, Campus Universitário de Santiago, Aveiro, Portugal; 2 CESAM, University of Aveiro, Campus Universitário de Santiago, Aveiro, Portugal; 3 Nuclear and Technological Institute (ITN) Department of Radiological Protection and Nuclear Safety, Sacavém, Portugal; 4 Department of Geosciences, University of Aveiro, GeoBioTec Research Center, Campus Universitário de Santiago, Aveiro, Portugal; 5 Department of Biology, Faculty of Sciences of the University of Porto, Porto, Portugal; 6 Interdisciplinary Centre of Marine and Environmental Research (CIIMAR/CIMAR), University of Porto, Porto, Portugal; University of Kansas, United States of America

## Abstract

In order to regulate the management of contaminated land, many countries have been deriving soil screening values (SSV). However, the ecotoxicological data available for uranium is still insufficient and incapable to generate SSVs for European soils. In this sense, and so as to make up for this shortcoming, a battery of ecotoxicological assays focusing on soil functions and organisms, and a wide range of endpoints was carried out, using a natural soil artificially spiked with uranium. In terrestrial ecotoxicology, it is widely recognized that soils have different properties that can influence the bioavailability and the toxicity of chemicals. In this context, SSVs derived for artificial soils or for other types of natural soils, may lead to unfeasible environmental risk assessment. Hence, the use of natural regional representative soils is of great importance in the derivation of SSVs. A Portuguese natural reference soil PTRS1, from a granitic region, was thereby applied as test substrate. This study allowed the determination of NOEC, LOEC, EC_20_ and EC_50_ values for uranium. Dehydrogenase and urease enzymes displayed the lowest values (34.9 and <134.5 mg U Kg, respectively). *Eisenia andrei* and *Enchytraeus crypticus* revealed to be more sensitive to uranium than *Folsomia candida*. EC_50_ values of 631.00, 518.65 and 851.64 mg U Kg were recorded for the three species, respectively. Concerning plants, only *Lactuca sativa* was affected by U at concentrations up to 1000 mg U kg^1^. The outcomes of the study may in part be constrained by physical and chemical characteristics of soils, hence contributing to the discrepancy between the toxicity data generated in this study and that available in the literature. Following the assessment factor method, a predicted no effect concentration (PNEC) value of 15.5 mg kg^−1^_dw_ was obtained for U. This PNEC value is proposed as a SSV for soils similar to the PTRS1.

## Introduction

Uranium (U) is a natural soil component, being originated from rocks in the Earth’s crust, where it mainly occurs in the form of oxides. Natural processes acting on rocks and soils, such as wind, water erosion, dissolution, precipitation and volcanic activity contribute for U dispersal in the environment [1]. The use of U as fuel in nuclear power plants has driven to its large-scale exploration worldwide. The U exploration became significantly important in the world during the Second World War, and later on during the Cold War, in both cases to supply military needs of the greatest potencies. Recently, the World Nuclear Association estimated worldwide reserves of U at 5.4 million tons in 2009, of which Australia had about 31%, followed by Kazakhstan (12%), Canada and Russia with 9% (http://www.world-nuclear.org/info/inf75.html). The remarkable energy crisis that is currently faced worldwide due to the exhaustion of carbon based energy resources is demanding further extraction of U, as nuclear energy arises as a potential solution. Hence, it is expected that the mining and milling of U will increase in the next decades, contributing for its widespread in the environment [2].

During the last century, Portugal has actively explored radioactive ores and was for some time ranked as one of the main U producers. The extraction of U ore in Portugal started in 1908, first driven by the interest in radium (being U a by-product) and then by the interest in its military applications, till 2001 [3], [4]. Most of the old U mines were located in the granitic regions of the Iberian Meseta, in the centre-north of Portugal (Beiras), [5]. Nowadays, although the mining activities ceased, like in several other places in the world, the old U mines represent a serious environmental problem, due to waste accumulation (mainly tailings and sludge) and improper disposal of radioactive material, composed by U and its daughter radionuclides [1], [5]–[16]. Soils and water are the two major environmental matrices affected by U contamination.

U has a long half-life, persisting in nature as different isotopes, with different chemical and radiological characteristics [17]. The toxic effects induced by this metal are caused by both properties. However, since U isotopes mainly emit alpha particles, with little penetration capacity, the main radiation hazards only occur after ingestion or inhalation of these isotopes and daughter radionuclides [17]. Once in the soil, U interacts with all the components of this matrix, such as clay minerals, aluminum and iron oxides, organic matter and microorganism, in a very complex system, where pH and organic matter seem to have the major role in controlling U mobility (pH 6) and leaching (pH<6) [18]. The high mobility/availability of U will in turn increase the ecological risks posed to soil and water compartments [19]–[27].

The soil has been recognized as an important compartment that provides crucial ecosystem services (e.g., filtering of contaminants, reservoir of carbon and a bank of genes) and is the support of agro-sylvo-pastoral production [28], [29] and of several other human activities. The soil compartment offers raw materials (e.g., peat, clay, ore) and contributes for climate regulation and biodiversity conservation, as well as other cultural services [30], [31]. The recognition of the importance of maintaining the provision of such services has increased the necessity to create appropriate legal tools to correctly and effectively protect and manage this resource. In this sense, the Soil Framework Directive proposed by the Commission of the European Communities (CEC), aims to establish a common strategy for the protection and sustainable use of soils [32]. For that end, this proposal defines measures for the identification of the main problems faced by soils, the adoption of strategies to prevent their degradation, as well as for the rehabilitation of contaminated or degraded soils [33]. The Soil Framework Directive will fill in the gap regarding soil protection, since this compartment has never been a target of specific protection policies at the European Community level [32]. Many countries, committed in regulating the management of contaminated land, have adopted generic quality standards, the soil screening values (SSVs) [34]. SSVs are concentration thresholds above which, more site-specific evaluations are required to assess the risks posed by soil contamination [35]. The SSVs should provide a level of protection to terrestrial species and ecological functions of the soil [35]–[37]. SSVs are particularly useful for the first tier of Ecological Risk Assessment (ERA) processes applied to contaminated sites, supporting the decision-making at this initial stage of assessment [38], which at the end is aimed in setting priorities for remediation and risk reduction measures [39]. In the case of Portugal, SSVs for soils have never been established for metals or organics. Only threshold concentrations of metals on sewage sludge were legally established to regulate the application of this solid waste on agricultural soils [40]. However, they are not appropriate for soil ERA purposes.

The use of natural reference soils in ecotoxicological tests has been recommended by several authors [41]–[43]. This is because the properties of the OECD artificial soil are not representative of the great majority of natural soils [44]. Different levels of toxicity, for each contaminant, can be expected in soils with different properties [45]–[48], hence it is important each country derives their own SSVs using natural reference soils representing the main types of soils within their territories. In this context, the main aim of this work was to obtain ecotoxicological data for U, performing soil enzymes activity tests, invertebrates and plant tests, using for that a Portuguese natural reference soil (PTRS1), that represents one of the dominant types of soil from a granitic region (cambisol) of the country [49]. As a result, enough data are gatheredas to make the first proposal of a SSV for this metal.

## Materials and Methods

The present study used a natural soil that was collected in a non-protected area, requiring no specific permission for its collection. Further, no work with endangered species was performed, and no vertebrate species were used in the ecotoxicological assays. Only tests with invertebrates and plants were performed. The invertebrates were obtained from laboratorial cultures maintained by the authors of this manuscript and plant seeds were obtained from a local supplier.

### 1. Test soil

The natural soil (PTRS1) used as test substrate in this study was collected in Ervas Tenras [Pinhel, Guarda, Portugal center; geographical coordinates: 40°44′4.27″N and 7°10′54.3″W), at 655 m altitude, in a granitic region.

A composite soil sample was collected and immediately brought to the laboratory where it was air dried. Another portion of the soil, was immediately sieved through a 2 mm mesh size and the sieved fraction (<2 mm) was stored in polyethylene bags, at −20°C, until further analysis of soil microbial parameters, which were performed within the period of one month. For the tests with soil organisms and plants, the soil was passed through a 4 mm mesh sieve and the sieved fraction (<4 mm) was defaunated through two freeze–thawing cycles (48 h at −20°C followed by 48 h at 25°C), before the beginning of the assays.

The physical and chemical properties (including total metal contents) of the PTRS1 soil were presented in a preliminary study by Caetano et al. [49], aimed in characterizing this soil as a reference substrate for ecotoxicological purposes. The main properties of the PTRS1 are also described in Tables 1 and 2. Briefly, soil-KCl 1 M and soil-deionized water suspensions (1∶5 m/v) were used for pH (KCl, 1 M) and pH-H_2_O measurements, respectively, according to ISO 17512–1 [50]- After 15 min of magnetic stirring and 1 h resting period, the pH of the suspension was measured using a WTW 330/SET-2 pH meter. A soil water suspension (1∶5 w/v) was used for the measurement of soil conductivity [51] Ten grams of PTRS1 were mechanically shaken in polypropylene flasks with 50 ml with deionized water filtered in a Milli-Q equipment (hereinafter referred as deionized water), water for 15 min. The mixture was left to rest overnight for soil bulk settling [51]. The conductivity of the resulting suspension was measured using an LF 330/SET conductivity meter. Soil water content was determined from the loss of weight after drying at 105°C, for 24 h. Organic matter (OM) content was determined by loss of ignition of dried soil samples at 450°C during 8 h [52]. For determination of water holding capacity (WHC) polypropylene flasks were prepared with a filter paper-replaced bottom, which after being filled up with soil samples, were immersed in water for 3 h. After this period, samples were left for water drainage during 2 h and the WHC was determined accounting to the loss of weight after drying at 105°C until weight stabilization [50].

**Table 1:** Physical and chemical properties of PTRS1 soil (retrieved from Caetano et al. [49]).

	pH(H_2_O)	pH(KCI,1 M)	Conductivity(mS cm^−1^)	OM (%)	WHC (%)		Particle-size distribution (%)			[U] (mg Cu kg^−1^ soil_dw_)
						Clay(<4 µm)	Silt(4–63 µm)	Sand(63 µm–2 mm)	Gravel(>2 mm)	
PTRS1	5.9±0.09	4.3±0.02	4.8±0.23	6.5±0.004	23.9±1.84	3.3	22.8	46.9	23.9	9.0

pH (H_2_O), pH (KCI, 1 M), OM (organic matter), and WHC (water holding capacity) are represented as average ± STDEV.

**Table 2:** Pseudo-total concentrations (mg/kg) of metals recorded in PTRS1 soil (average ± standard deviation) extracted with aqua régia, (retrieved from Caetano et al.[49]).

Metal	PTRS1
Ag	0.1±0.0
Al	25628.5±5130.0
B	2.2±0.8
Ba	45.8±8.0
Be	1.2±0.2
Cd	0.1±0.1
Co	5.6±1.1
Cr	10.8±2.1
Cu	9.0±1.8
Fe	24921.4±4534.4
Li	124.4±22.9
Hg	5253.5±1025.5
Mn	386.8±77.9
Mo	0.9±0.2
Na	78.1±14.9
Ni	4.6±0.9
Pb	12.5±2.2
Sb	0.2±0.0
Sn	10.4±1.9
U	7.8±1.7
V	37.8±14.1
Zn	57.1±8.9

### 2. Test substance

For all the test organisms, the natural soil was spiked with a stock solution of uranyl nitrate 6-hydrate, UO_2_(NO_3_)_2_6H_2_O (98%, PANREAC) prepared with deionized water in order to obtain a range of concentrations, which were ascertained by range finding tests performed with the different test species.

For soil enzyme tests, the PTRS1 soil was spiked with the following concentrations: 0.0, 134.6, 161.5, 193.8, 232.5, 279.0, 334.8, 401.8, 482.2, 578.7, 694.4, 833.3, 1000 mg U kg^−1^_dw_. To obtain these concentrations, the stock solution of uranyl nitrate was diluted in the volume of deionized water required to adjust the soil moisture at 80% of its maximum water holding capacity (WHC_max_).

The following U concentrations were used to expose the earthworms in the reproduction tests: 0.0, 113.1, 124.4, 136.9, 150.5, 165.6, 231.9, 324.6, 454.5, 500.0, 550.0, 605.0, 665.5 mg U kg^−1^_dw_. For potworms, collembolans and terrestrial plant assays the same range of concentrations was tested: 0.0, 167.4, 192.5, 221.4, 254.6, 292.7, 336.6, 420.8, 526.0, 657.5, 756.1, 869.6, 1000 mg U kg^−1^_dw_.

The volume of deionized water required to adjust the WHC of the soil to a given percentage of its maximum value was used to dilute the stock solution for these tests. After spiking the soil was left to rest for equilibration for 48 h before testing.

### 3. Ecotoxicological assessment

#### 3.1 Soil microbial activity

For testing the effect of increasing concentrations of U on soil microbial parameters, a 30-day exposure was firstly conducted. Ten grams of sieved PTRS1 soil per replicate and concentration were spiked with different U concentrations, a total of three replicates were used per treatment. Six replicates with the same amount of soil only moistened with deionized water were also prepared for the control. The soil was incubated at 20±2°C and a photoperiod of 16 h^L^:8 h^D^. During the incubation period, the soil moisture was weekly monitored by weighing the pots, and whenever needed it was adjusted to 80% of its WHC_max_ by adding deionized water. At the end, 1 g of each replicate from the control and concentrations tested was stored in individual falcon tubes at −20°C for approximately one month. Thereby, a total of 9 sub-replicates were made for each concentration. The soil was thawed at 4°C before analysis.

The biochemical parameters analyzed were: the activity of arylsulphatase, dehydrogenase, urease, and cellulase enzymes and changes in the nitrogen mineralization (N mineralization) and potential nitrification.

For the determination of arylsulphatase activity, the method proposed by Tabatabai and Bremner [53] and Schinner et al. [54] was followed. After addition of 1 mL of p-nitrophenylsulfate (0.02 M), soil sub-samples were incubated for one hour, at 37°C. The nitrophenyl liberated by the activity of arylsulphatase was extracted and colored with a 4 mL of sodium hydroxide (0.5 M) and determined photometrically at 420 nm. The results were expressed as µg p-nitrophenylsulfate (p-NP) g^−1^ soil _dw_ h^−1^.

The method proposed by Öhlinger [55] was used to assess the dehydrogenase activity. The samples were suspended in 1 mL of trifeniltetrazol chloride (TTC) (3.5 g L^−1^) and incubated at 40°C for 24 h. The triphenylformazan (TPF) produced was extracted with acetone and measured spectrophotometrically at 546 nm. The results were expressed as µg TPF g^–1^ soil_dw_ h^–1^.

The cellulase activity was tested according to the method proposed by Schinner et al. [54] and Schinner and von Mersi [56]. The reducing sugars produced during the incubation period, after addition of 1.5 mL of acetate buffer (2 M), caused the reduction of hexacyanoferrate (III) potassium to hexacyanoferrate (II) potassium in an alkaline solution. This last compound reacts with ferric ammonium sulfate in acid solution to form a ferric complex of hexacyanoferrate (II), of blue colour, which is colorimetrically measured at 690 nm and expressed as µg glucose g^−1^ soil_dw_ 24 h^−1^.

N mineralization activity was measured according to Schinner et al. [54]. For this purpose the soil samples were incubated for 7 days at 40°C. During this period, the organic forms of N were converted to inorganic forms (mainly ammonium ion, NH_4_^+^), which were determined by a modification of the Berthelot reaction, after extraction with 3 mL of potassium chloride (2 M). The reaction of ammonia with sodium salicylate in the presence of sodium dichloroisocyanurate formed a green colored complex in alkaline pH that was measured at 690 nm and expressed as µg N g^−1^ soil_dw_ d^−1^.

The urease activity was assayed according to the method proposed by Kandeler and Gerber [57] and, Schinner et al. [54]. The samples were incubated for 2 h at 37°C after the addition of 4 mL of a buffered urea solution (720 mM). The ammonia released was extracted with 6 mL of potassium chloride (2 M) and determined by the modified Berthelot reaction. The quantification was based on the reaction of sodium salicylate with ammonia in the presence of chlorinated water. UR was detected at 690 nm and expressed as µg N g^−1^ soil_dw_ 2 h^−1^.

The quantification of potential nitrification was determined by the method of Kandeler [58], which is a modification of the technique proposed by Berg and Rosswall [59]. The ammonium sulphate (4 mL, 10 mM) was used as substrate, and soil samples were incubated for 5 h, at 25°C. Nitrate released during the incubation period was extracted with 1 mL of potassium chloride (2 mM) and determined colorimetrically at 520 nm. This reaction was expressed as µg nitrite (N) g^–1^ soil_dw_ h^–1^.

#### 3.2. Invertebrate and plant tests. Test organisms and culture conditions:

The earthworm *Eisenia andrei* (Oligochaeta: Lumbricidae), the potworm *Enchytraeus crypticus* (Oligochaeta: Enchytraeidae) and the springtail *Folsomia candida* (Collembola: Isotomidae) were used as invertebrate test organisms. All organisms were obtained from laboratorial cultures, kept under controlled environmental conditions (temperature: 20±2°C; photoperiod: 16 h^L^:8 h^D^). The earthworms (*E. andrei*) are maintained in plastic boxes (10 to 50 L) containing a substrate composed by peat, dry and defaunated horse manure (through two freeze–thawing cycles (48 h at −20°C followed by 48 h at 65°C), and deionized water. The pH of the culture medium is adjusted to 6.0–7.0 with CaCO_3_. The organisms are fed, every 2 weeks, with six table spoons of oatmeal previously hydrated with deionized water and cooked for 5 min. The potworms (*E. crypticus*) are cultured in plastic containers (25.5 cm length; 17.4 cm width; 6.5 cm height), which are filled with pot soil moistened to the nearest 60% of its WHC_max_ and with pH adjusted to 6.0±0.5. The organisms are fed twice a week with a tea spoon of macerated oat. The collembolans (*F. candida*) are maintained in plastic containers filled with culture medium composed by moistened Plaster of Paris mixed with activated charcoal 8∶1 (w:w). They are fed with half of a tea spoon of granulated dry yeast, twice a week. The food is added in small amounts to avoid spoilage by fungi.

Seeds from four plant species (two dicotyledonous and two monocotyledonous), purchased from a local supplier, were used for seed germination and growth tests: *Avena sativa, Zea mays*, *Lacuta sativa* and *Lycopersicon esculentum.*

#### Reproduction tests with invertebrates:

Previous studies from our team, at least with earthworms from the same laboratorial cultures, have proved that these organisms were not exposed to meaningful levels of metals (especially U, in laboratorial culture conditions) [60]. The accomplishment of validity criteria, by all the controls of the assays (herein described) with the three invertebrate species, also confirmed that the test animals were not previously exposed to toxic levels of metals through test containers, substrates or food. The reproduction tests with *E. andrei*, *E. albidus* and *F. candida* were carried out according to the ISO guidelines 11268-2 [61], 16387 [62] and 11267 [63], respectively. Each replicate of the invertebrate tests contained 10 individuals in a certain developmental stage: the earthworms had a fully developed clitellum and an individual fresh weight between 250 and 600 mg; the potworms were 12-mm size; and the springtails were 10–12 days old. Five hundred grams of dry soil were weighted per test vessel for earthworms. For the tests with potworms and collembolans 20 g and 30 g of soil were weighted per replicate, respectively. Following an ECx sampling design, which considers more concentrations and less number of replicates, two replicates per concentration and five replicates for the control were prepared in the reproduction tests with *E. andrei*. Adult earthworms were removed from the test containers after 28 days. The produced cocoons persisted in the soil until 56 days have been completed. After this period, the juveniles from each test container were counted. During the test, organisms were fed once a week, with 5 g per box of defaunated horse manure (using the same procedure above described), and the soil moisture content was weekly monitored (following the procedures outlined in ISO guideline 11268-2 [61]).

The *E. albidus* reproduction test was held for 28 days and the adults were left in the vessels until the end of the test. About 2 mg of rolled oats were placed on the soil surface, weekly to feed the animals. At the end of the test, the potworms were killed with alcohol, colored with Bengal red and counted according to the Ludox Flotation Method, as described in ISO 16387 [62]. The reproduction tests with *F. candida* took four weeks to be completed. The collembolans were fed with granulated dry yeast, obtained from a commercial supplier, being weekly added (about 2 mg of yeast per test vessel) to the soil surface. At the end of the test, the containers were filled with water and the juveniles were counted after flotation. The addition of a few dark ink drops provided a higher contrast between the white individuals and the black background. The organisms were then counted through the use of the ImageJ software (online available: http://rsb.info.nih.gov/ij/download.html). The exposure was carried out at 20±2°C and a photoperiod of 16^L^:8^D^. For both species five replicates of uncontaminated natural PTRS1 soil were prepared for the control. The same ECx sampling design applied for earthworms was followed. However, in order to reduce the variability of the results, three replicates were prepared per test concentration (instead of two for the earthworms).

#### Seed germination and plant growth tests:

Germination and growth tests with terrestrial plants were performed following standard procedures described by the ISO guideline 11269-2 [64]. For this purpose, 200 g_dw_ of the spiked soil with the concentrations described above was placed in plastic pots (11.7 cm diameter, 6.2 cm height) and tested. In this case, the amount of water required to adjust the WHC_max_ of the soil to 45% was used to dilute the stock solution and to moist the soil at the beginning of the test. The soil was placed in the plastic pots (11.7 cm diameter, 6.2 cm height). In the bottom of each plastic pot a hole was previously made to let a rope passing through, hence allowing communication with the pot below that was filled with deionized water. After soil spiking and soil saturation with water twenty seeds were added to each pot and gently covered with the spiked soil. The level of water in the lower recipient was adjusted whenever needed, as to guarantee the necessary conditions of moisture according to, the recommendations specified in [64]. Five replicates of uncontaminated natural PTRS1 soil were prepared for the control, while three replicates were tested per concentration, in order to minimize the variability of the results, and to follow the ECx sampling design, similarly used for the invertebrate tests.

At the beginning of the test, nutrients (Substral - Plants fertilizer using 1 bottle cap for 2 L of water proportion according to the manufacturer recommendation; Fertilizer NPK: 6-3-6; nitrogen (N): 6%; phosphate (P_2_O_5_): 3%; potassium (K_2_O): 6%; iron (Fe): 0,03%; trace elements: Cu, Mn, Mo and Zn), were added in each lower recipient containing the water. Pots were maintained at constant conditions of temperature (20±2°C), photoperiod (16 hL: 8 hD) and light intensity (25.000 lux). Daily observations were carried out to record the number of emerged seeds. Only the first five emerged seeds were left to grow, the remaining ones were counted and harvested. Fourteen days later, the assay was finished and the fresh and dry biomass above soil was assessed for each test species at the end of the exposure period.

The endpoints seed germination, and fresh and dry biomass, above soil, were assessed for each species at the end of the exposures according to the methods outlined in ISO guideline 11269-2 [64].

For this work, a battery of enzymes involved in different biogeochemical cycles S (sulfur cycle), N (Nitrogen cycle), C (Carbon cycle), as well as enzymes more indicative of the good physiological conditions of the whole microbial community (e.g. dehydrogenases) were selected. The species of invertebrates and plants were selected based on the availability of standard protocols. Since we aimed to obtain data for the derivation of SSVs, for regulatory purposes, this procedure is recommended.

## Statistical Analysis

A one-way analysis of variance (one-way ANOVA) was performed to test significant differences between the uranium concentrations tested for each endpoint analyzed: the activity of enzymes, the number of juveniles produced by potworms and collembolans, the number of emerged seeds, and the fresh and dry mass of the plants. The Kolmogorov-Smirnov test was applied to check data normality, whereas homoscedasticity of variances was checked by the Levene’s test. When these two assumptions of the one-way ANOVAs were not met, a Kruskal-Wallis analysis was performed. The statistical analysis was run in the SigmaPlot 11.0 software for Windows. When statistical significant differences were recorded, the Dunnett's (for parametric one-way ANOVA) or the Dunn’s test (for non-parametric ANOVA) was carried out to perceive which concentrations were significantly different from the respective control. Based on the outcomes of the multiple comparison tests the NOEC (no-observed-effect-concentration) and LOEC (low-observed-effect-concentration) values were determined. The EC_20_ and EC_50_ values for each endpoint were calculated whenever possible, after fitting the data to a log-logistic model using the STATISTICA 7.0 software.

## PNEC-Based SSV Derivation

Following the approach suggested by the Technical Guidance Document published by the European Commission [65], a predicted no effect concentration (PNEC) for U in the PTRS1 soil was determined, based on the assessment factor method For that, it was by used the lowest point estimate (i.e., NOEC and EC_20_ values) and applied the appropriate assessment factor based on the criteria of the Guidance Document [65]. The lowest point estimate calculated was for arylsulphatase activity. Considering that more than three NOEC values were obtained in this study, for at least three species, an assessment factor of 10 was applied, The PNEC value was calculated through the application of the following equation:
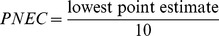



## Results and Discussion

### 1. Soil microbial activity

As far as authors are aware, this study is one of the few studies gathering data regarding the ecotoxicity of spiked soils with U on soil microbial parameters. Only a study from Sheppard et al. [66] has analyzed the effect of U on soil phosphatase activity in eleven different Canadian soils (including an agricultural, a boreal forest and a garden soil). This study recorded a significantly depressed activity only at the highest concentration tested (1000 mg U kg soil_dw_^−1^) for all the soils. These results suggested that probably, soil phosphatase activity was one of the less sensitive soil microbial parameters to U. In fact, Pereira et al. [7] also reported the low sensitivity of this parameter in mine soils contaminated with metals.

**Figure 1:**
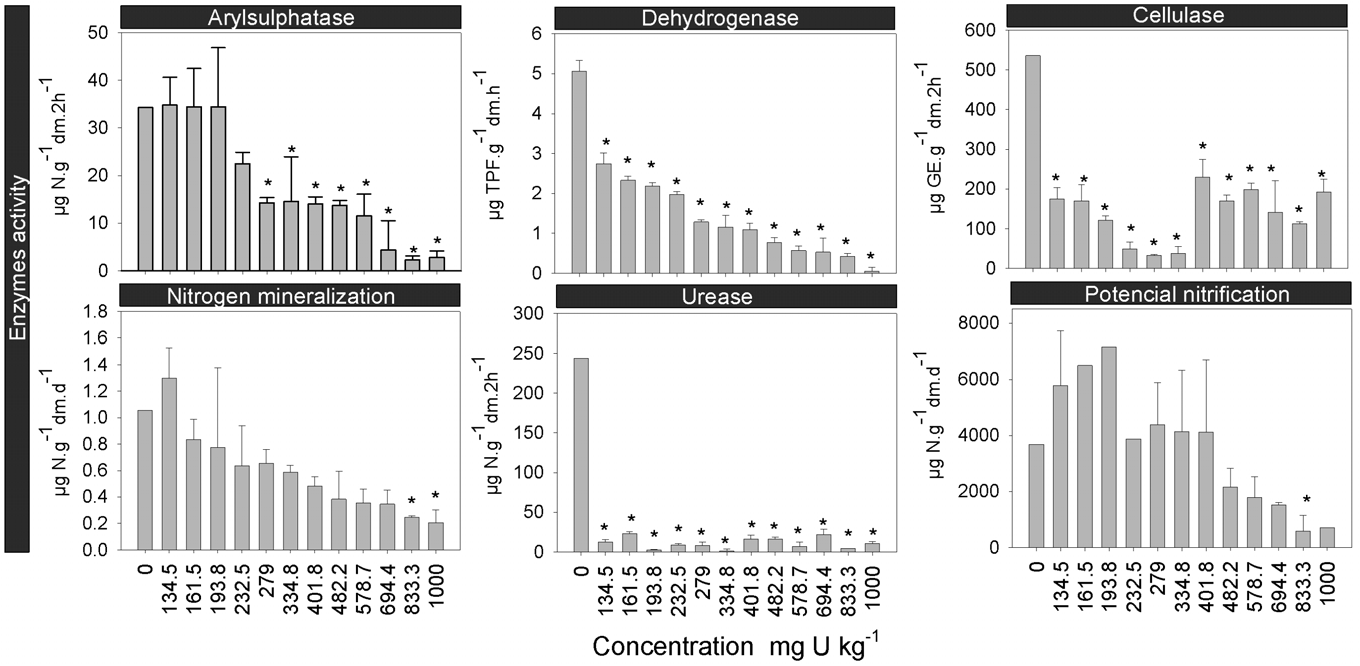
Soil enzyme activities, N mineralization and potential nitrification. Response of the arylsulphatase, dehydrogenase, cellulase urease, activity, N mineralization and potential nitrification to soils spiked with a range of uranium concentrations. The error bars indicate the standard deviation. The asterisks point out significantly differences from the control (P<0.05).

The variation in soil enzyme activities, N mineralization and potential nitrification in the PTRS1 soil, spiked with different U concentrations, is shown in Figure 1, and the Table 3 summarizes the toxicity values obtained for each biochemical parameter.

**Table 3:** Toxicity data obtained for copper (mg U kg^−1^ soil_dw_) in PTRS1 soil on soil microbial processes, invertebrates and plants.

Biota	Endpoint	NOEC	LOEC	EC_20_	EC_50_
**Microbial** **parameters**					
Arylsulphatase		232.5	279	155.3 (84.76–255.87)	295.6 (216.09–375.17)
Dehydrogenase		<134.5	≤134.5	34.9 (20.52–59.35)	110.3 (83.25–137.47)
Nitrogenmineralization	Enzyme activity	694.4	833.3	152.2 (46.66–257.79)	347.0 (211.25–482.91)
Celulase		≤134.5	≥134.5	n.d.	n.d.
Urease		<134.5	≤134.5	<134.5	<134.5
Potencialnitrification		<134.5	≤134.5	429.5 (229.53–629.46)	610.0 (459.07–761.11)
**Invertebrates**					
* Eisenia andrei*	Rep. (56 days)	500.0	550.0	474.8 (391.47–558.04)	631.0 (532.78–699.21)
* Enchytraeus* *crypticus*	Rep. (28 days)	420.8	526.0	469.7 (355.47–584.04)	518.6 (480.40–556.90)
* Folsomia candida*	Rep. (28 days)	675.5	756.1	343.4 (172.23–514.60)	851.64 (606.10–1097.18)
**Plants**					
* Avena sativa*	Germination	≥1000	>1000	n.d.	n.d.
* Zea mays*	Germination	≥1000	>1000	n.d.	n.d.
* Lactuca sativa*	Germination	≥1000	>1000	n.d.	n.d.
* Lycopersicon* *esculentum*	Germination	≥1000	>1000	n.d.	n.d.
* Avena sativa*	Fresh mass	≥1000	>1000	n.d	n.d.
* Zea mays*	Fresh mass	≥1000	>1000	n.d	n.d.
* Lactuca sativa*	Fresh mass	≥1000	>1000	n.d	n.d.
* Lycopersicon* *esculentum*	Fresh mass	≥1000	>1000	n.d	n.d.
* Avena sativa*	Dry mass	≥1000	>1000	n.d.	n.d.
* Zea mays*	Dry mass	≥1000	>1000	n.d.	n.d.
* Lactuca sativa*	Dry mass	<167.4	≤167.4	n.d.	n.d.
* Lycopersicon* *esculentum*	Dry mass	≥1000	>1000	n.d.	n.d.

For ECx point estimates the 95% confidence limits are presented in brackets. n.d.- not determined; Rep. – reproduction.

U had a clear inhibitory effect in almost all functional parameters tested. Overall, dehydrogenase and urease were the most affected soil enzymes by U, being their activity significantly inhibited at concentrations equal or lower than 134.5 mg U kg soil_dw_^−1^ (Table 3). Dehydrogenases have a relevant role in the oxidation of soil organic matter (SOM), being a good indicator of the active microbial biomass in the soil compartment [67]. As such, U (in the form of uranyl) strongly affected the normal microbial activity in PTRS1 soil. Meyer et al. [68] also observed a significant reduction in respiration rates of a soil exposed to depleted uranium (DU), but only for concentration equal and higher than 500 mg U kg soil_dw_^−1^. Indeed, the inhibition of urease activities indicates that U had a deleterious effect on soil N-cycle (Figure 1, Table 3). The reduction in the activity of this enzyme may have been caused by a negative effect of U on the overall microbial biomass, which in turn was translated into a reduction in the oxidation rate of organic N into ammonium [58], [69]. Arylsulphatase is regularly involved in the S-cycle by catalyzing hydrolysis reactions in the biogeochemical transformation of S [67]. This parameter was significantly affected by U at a LOEC of 279.0 mg U kg soil_dw_^−1^. On its turn, the cellulase activity was significantly inhibited at intermediate U concentrations. However in the highest concentrations the tendency was reversed and the activity increased, but not for levels significantly different from the control (Figure 1). Thereby, we can conclude that the C-metabolism associated with the degradation of SOM and catalyzed by these extracellular enzymes [70] was constrained by U. N mineralization and potential nitrification are indicators of the functioning of the N-cycle, hence providing an overview of the activity of specific microbial groups (nitrifying bacteria) directly involved in both processes [71]. The general pattern of response observed for these two parameters corresponded to stimulation at the lower U concentrations and inhibition under the highest ones (Figure 1), leading to EC_50_ values of 347.0 and 610.0 mg U kg soil_dw_^−1^ (Table 3), respectively. It has been stated that N mineralization is normally less sensitive than potential nitrification, since the former is carried out by a wider diversity of microorganisms [71]. However, our data showed the opposite (Figure 1). Meyer et al [68] did not observe effects on nitrogen mineralization of the test soil for U concentrations up to 25000 mg kg soil_dw_^−1^, however the form of U tested by these authors (schoepite UO2(OH_2_).H_2_O) was less soluble than the one tested in this soil.

The sensitivity of soil microbial parameters to metals has already been demonstrated by several authors, either in metal-polluted or in artificially spiked soils (e.g.,[4], [72]–[77]). Dehydrogenase and urease had generally been referred as the most affected enzymes for different metals (e.g., Cu, Pb, Zn, Cd, Fe, Cr, Ni), (e.g.,[72], [73], [78], [79]). Arylsulphatase and cellulase, however, have shown contradictory responses in different studies. Some authors observed negative correlations between arylsulphatase and cellulase activities and Zn [75] and Cu concentrations, respectively [80], [81]; while others observed positive correlations between arylsulphatase and Cd [81], and no changes on cellulase activities in the presence of metals in urban soils was observed [82]. Usually, potential nitrification is negatively influenced by the presence of metals and metalloids such as Pb, Cu and As [7], [81]; and the inhibitory effect of some metals (like Zn, Cd and Pb) on N mineralization was also reported by Dai et al. [83]. Antunes et al [81] found negative correlations (based on the Spearman coefficient) between U levels in soils from an abandoned U mine (presenting a mixture of metals) and the activities of urease and cellulase enzymes. For dehydrogenase, potential nitrification and arylsulphatase no significant correlations were detected. Nevertheless, this study analyzed mine contaminated soils, where the mixture of metals, may cause either synergistic or antagonistic effects, and where a well adapted and functional microbial community was likely established.

The inhibition of soil enzyme activities recorded could have been caused by toxicological effects of metals on soil microorganisms with subsequent decrease in their abundance and/or biomass; and/or by the direct inactivation of extracellular enzymes by metals [84]. Notwithstanding, the levels of metals may be not the sole effect on soil microbial activity. Soil properties (e.g., pH, organic matter content, nutrients and soil texture) may also interfere and modulate the bioavailability and toxicityof metals on soil enzymes [74], [85]. Clays can retain and protect extracellular hydrolases, namely urease [73]. But the low clay content of PTRS1 soil (3.32%) (Table 1) might have increased U bioavailability, leading to the impairment of soil microbial community through cytotoxic effects, hence reducing their metabolic activity [81]. Additionally, the low pH of PTRS1 soil (Table 1) might have contributed for U availability and impacts on enzyme processes, potential nitrification and N mineralization, particularly at higher U concentrations, as previously observed by Coppolecchia et al. [75] for arylsulphatase in the presence of Zn and low pH.

The above results illustrated well the effects of U in the performance of soil enzymes, reinforcing the importance of these parameters as bioindicators of soil quality. Indeed, the EC_20_ values calculated for dehydrogenase (34.9 mg Ukg soil_dw_^−1^), urease (<135.5 mg Ukg soil_dw_^−1^), N mineralization (152.2 mg Ukg soil_dw_^−1^) and arylsulphatase (155.3 mg Ukg soil_dw_^−1^) are within the environmental concentrations quantified in soils from an abandoned U mine, following extractions with *aqua regia* or with rainwater [8]. In this sense, the data herein generated represent a great asset for the derivation of SSVs, since they have a great ecological representativeness.

### 2. Uranium toxicity to the reproduction of soil invertebrates

The reproduction tests with the three invertebrate species revealed that *E. andrei, E. crypticus and F. candida* were quite sensitive to U in the PTRS1 soil. Tests fulfilled the validity criteria established by the standard guidelines for control replicates [61]–[63]. The resulting NOEC, LOEC, EC_20_ and EC_50_ values obtained in this study and toxicity data available in the literature are summarized in the Table 3.

**Figure 2:**
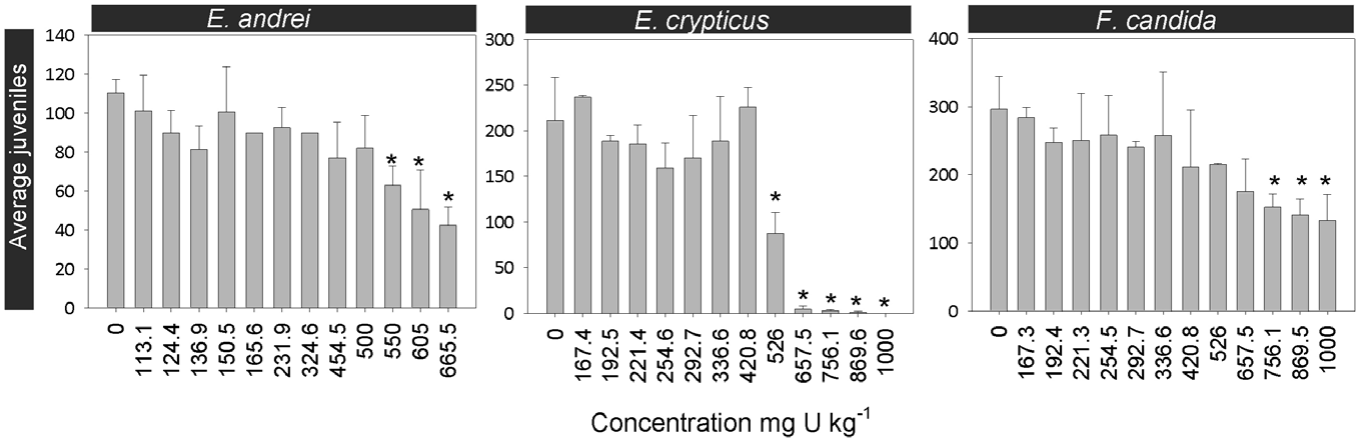
Reproduction of invertebrates. Results obtained exposing *Eisena andrei*, *Enchytraeus crypticus* and *Folsomia candida*, to natural PTRS1 soil, contaminated with different concentrations of U. The error bars indicate the standard deviation. The asterisks point out significantly differences from the control (P<0.05).

The effects of U in the reproduction of *E. andrei* were evident, since statistical significant differences were found between the control and the highest tested concentrations of U for this organism (F = 5.218, d.f.  = 23, p = 0.002) (Figure 2). The tested metal did not significantly affect the reproduction of *E. andrei* at concentrations up to 500.0 mg U kg soil_dw_^−1^ (NOEC) but compromised this endpoint for concentrations above 550.0 mg U kg soil_dw_^−1^ (LOEC). EC_20_ and EC_50_ values of U for *E. andrei* reproduction were 474.83 mg U kg soil_dw_^−1^ and 631.00 mg U kg soil_dw_^−1^, respectively (Table 3). The results obtained in our study, did not support those of Sheppard and Stephenson [86] (Table 4) that did not record toxic effects for *E. andrei* below 1000 mg U kg soil_dw_^−1^ (soils (carbonated): pH 7.5, 18% organic matter, 18% clay). However, they found an inhibition of juveniles production in two soils spiked with U, presenting low organic matter (2.2% and 1%) and a pH of 7.5 and 6.2, respectively (Table 4). According to the literature, the adsorption of metals to soil components is dependent on its physical and chemical properties, therefore influencing their toxicity to soil organisms [41], [47], [48]. Chelinho et al. [87], observed that soils with an organic matter content below 4% reduced or completely inhibited earthworms reproduction. However, the PTRS1 natural soil, had a high organic matter content, 6.2% (according to the classification provided by Murphy et al. [88]). Besides, as previously checked, the intrinsic properties of this soil did not compromise the performance of earthworms [49]. A high organic matter content of soils is usually related with a decrease in the toxicity of the contaminants for the organisms [41], [43], [89]. However, this was not the case in the study. In fact, Lourenço [60], [90] exposed *E. andrei* to a U mine soil with 215.72±8.50 mg U kg soil_dw_^−1^, a pH of 7.79±0.01, and 7.71±0.60% of organic matter and observed that the bioaccumulation of U and daughter radionuclides was in tandem with loss of DNA integrity of coelomocyte cells, changes in the frequency of cells of immune system and also with histopathological changes (especially of the epidermis and chloragogenous tissue and intestinal epithelium). In fact, some other authors [91] had also suggested that the direct dermal exposure of earthworms to metals in the soil pore water, the ingestion of water, and/or soil particles may strongly favor the bioaccumulation of metals. Since pH is variable in the different compartments of gastrointestinal tract of earthworms, it can increase the mobilization of contaminants from soil after its ingestion [92], [93].

**Table 4:** Toxicity of copper (mg U kg^−1^soil_dw_) reported in the literature for the reproduction of soil invertebrates using different soil types with different physical and chemical characteristics.

Species	Endpoint	Soiltype	Physical-chemicalparameters			Point estimates(mg U kg^−1^soil_dw_)				Reference
			pH	OM (%)	Clay (%)	NOEC	LOEC	EC_20_	EC_50_	
		Canadian soil	6.2	1.0	2.0	n.d.	n.d.	>1000	n.d.	Sheppard and Stephenson [86]
*Eisenia fetida*	Rep. (56 days)	Canadian soil	6.2	1.0	2.0	n.d.	n.d.	>1120	n.d.	Sheppard and Stephenson [86]
		Canadian soil	7.5	2.2		>838	n.d.	n.d.	n.d.	Sheppard and sheppard [98]
		Canadian soil	7.5	18.4		>994	n.d.	n.d.	n.d.	Sheppard and sheppard [98]
*Folsomia Candida*	Rep. (28 days)	Canadian soil	7.5	2.2	24.0	n.d.	n.d.	840.0	n.d.	Sheppard and Stephenson [86]
		Canadian soil	7.5	n.d.	n.d.	n.d.	n.d.	>720	n.d.	Sheppard and Sheppard [98]
*Elymus lanceolatus*	Germination	Canadian soil	6.2	1	2	n.d.	>1000	n.d.	n.d.	Sheppard and Stephenson [86]
*Elymus lanceolatus*	Germination	Canadian soil	7.5	2.2	24	n.d.	>1001	n.d.	n.d.	Sheppard and Stephenson [86]
*Zea mays*	Dry mass	European soil	5.2	2.5	n.d.	n.d.	>100	n.d.	n.d.	Stojanović et al., [108]

OM - organic matter, Rep. - reproduction, n.d. - not determined., germ.- germination.

Although, other metals were present in the contaminated soil tested by Lourenço et al. [60], [90] U likely had a crucial role in the toxic effects observed, because it persisted in the whole body till 56 days. These authors suggested that the changes observed in DNA integrity were likely early warning indicators of effects on the growth and reproduction of the organisms. And in fact, effects on reproduction were observed in our study. Further, Giovanetti et al. [94]. exposed *E. fetida* natural U- and DU-contaminated soil (no information on soil type) for 7 and 28 days. Regarding natural U, no mortality or significant changes in weight were observed for both exposure periods up to 600 mg U kg^−1^_dw_. The chloragogeneous tissue, the main storage tissue of U, presented meaningful changes after 7 days of exposure for 300 mg U Kg^−1^, while DNA strand breaks increased in a dose dependent manner above 150 mg U kg soil_dw_^−1^.

Regarding *E. crypticus* reproduction, it was significantly not reduced above 526.0 mg U kg soil_dw_^−1^ (LOEC) (Table 3) (F = 31.05, d.f.  = 12, p<0.05). The EC_20_ and EC_50_ values estimated were respectively 469.7 and 518.6 mg U kg soil_dw_^−1^. Although no toxicity values are reported for the lowest concentrations tested, enchytraeids showed considerable sensitivity to U, since the number of juveniles was minimal or no juveniles were produced by *E. crypticus* at concentrations above 657.5 mg U kg soil_dw_^−1^ (Figure 2). Despite enchytraeids are commonly used in standardized toxicity tests, to the best of our knowledge, no data are available in the literature regarding the effects of U on the reproduction of this species. The available information concerns only the toxic effects caused by other metals or by natural soil properties in the reproduction of this species [43], [48], [95]–[97]. Thus, taking into account this literature review, pH and CEC were the most important parameters controlling the high sensitivity of enchytraeids to metals. Additionally, and according to Kuperman et al. [43], adults survival and juveniles production by *E. crypticus* can be maximized in natural soils with properties within the following ranges: 4.4–8.2 pH; 1.2–42% OM; 1–29% clay. The PTRS1 natural soil used as test substrate fell into in these ranges (Table 3), and similarly to *E. andrei,* the reproduction of this species was not compromised during the validation of the PTRS1 natural soil as a reference soil [49], meaning that the soil properties did not limit the performance of *E. crypticus*.

Concerning *F. candida*, U affected the production of juveniles, as shown by a significant decrease of this endpoint along the concentrations tested (F = 11.6, d.f.  = 12, p<0.05) (Figure 2). The number of juveniles was not significantly affected up to 675.50 mg U kg soil_dw_^−1^ (NOEC), but it was significantly decreased for U concentrations equal to or greater than 756.10 mg U Kg^−1^ (LOEC) (Table 3). The EC_20_ value estimated for reproduction was 343.41 mg U kg soil_dw_^−1^ which is considerably lower than the toxicity data reported by Shepard et al. [98], EC_20_>710 mg U kg soil_dw_^−1^, in two loam soils with pH 7.5 (Table 4). The low sensitivity of *F. candida* to U was also observed by Sheppard and Stephenson [86] which tested 3 soils amended with a range of U concentrations and aged for 10 years before testing. In this study, the lowest EC_20_ value obtained was 840 mg U kg soil_dw_^−1^ in a loam soil (pH 7.5, 24% clay, 2.2% OM) (Table 4). Despite this, *F. candida* was more sensitive in the study of Sheppard and Stephenson (since their EC_20_ value was similar to the EC_50_ recorded in our study 851.64 mg U kg soil_dw_^−1^). When considering the number of juveniles produced, U was less toxic to *F. candida* comparatively to *E. andrei* and *E. crypticus*. The lower sensitivity of *F. candida* is also consistent with other studies, when the effects of other metals in the reproduction of the three species was investigated [97], [99], or even when other species of collembolans were analyzed [86]. The exposure of *F. candida* to chemicals in soil is apparently lower than for earthworms, which are exposed both by ingestion of contaminated soil (mineral particles, organic matter and chemicals in the soil solution) and also through direct dermal contact [100]. Despite the widely known influence of soil parameters on the bioavailability of chemicals and their influence on the reproduction of soil organisms, less is known about the intrinsic effects of physicochemical parameters of the soils in the reproduction of *F. candida*. In general, several authors had reported a high tolerance of *F. candida* reproduction to a wide range of soil textural classes, organic matter contents and soil pH [48], [101], [102]. Once again the performance of this species was not compromised by the intrinsic properties of the PTRS1 soil. Hence, the effects observed can undoubtedly be attributed to U exposure.

### 3. Phytotoxicity of uranium

**Figure 3:**
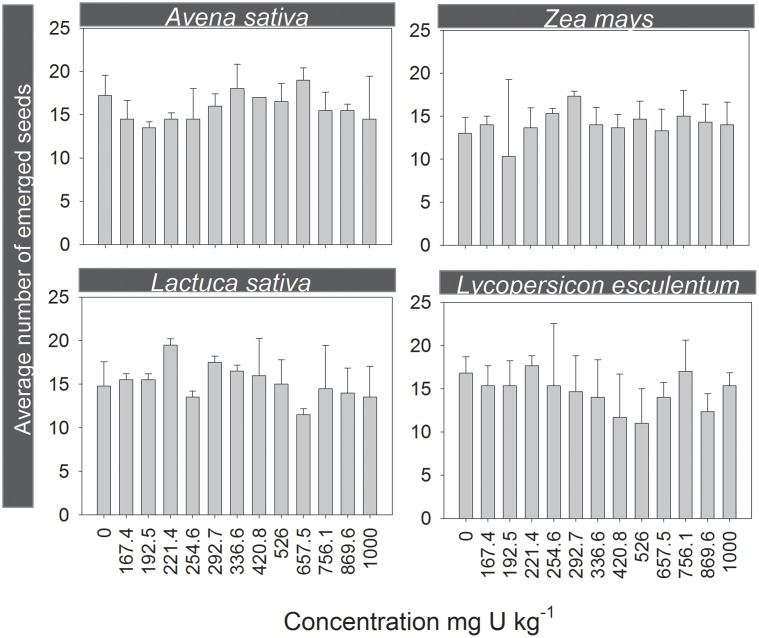
Seed germination of plants. Average number of emerged seeds in monocotyledonous, *Avena sativa and Zea mays* and in dicotyledonous species, *Lycopersicon esculentum and Lactuca sativa,* grown in PTRS1 soil contaminated with U. The error bars indicate the standard deviation.

Relatively to terrestrial plants tests, all the validity criteria as described by the standard guidelines were attained [64]. Data obtained showed no significant effects on seeds emergence for all species tested (p>0.05). In fact, it was observed a relatively high rate of germination, either in monocotyledonous and dicotyledonous species (Figure 3). This outcome was somewhat expected, based on previous studies (e.g.,[22]). Seed coats form a barrier which protects embryos from a wide range of contaminants, especially metals. Thus, the germination relies almost exclusively on the seed reserves making it a less sensitive endpoint to the toxicity of soil pollutants [103].

An apparent hormetic effect was recorded for the other endpoints measured for almost all plant species. Such occurrence was recorded by other authors and it was attributed to the use of U as uranyl nitrate, which corresponds to a supplementary dose of N given to plants [98].

**Figure 4:**
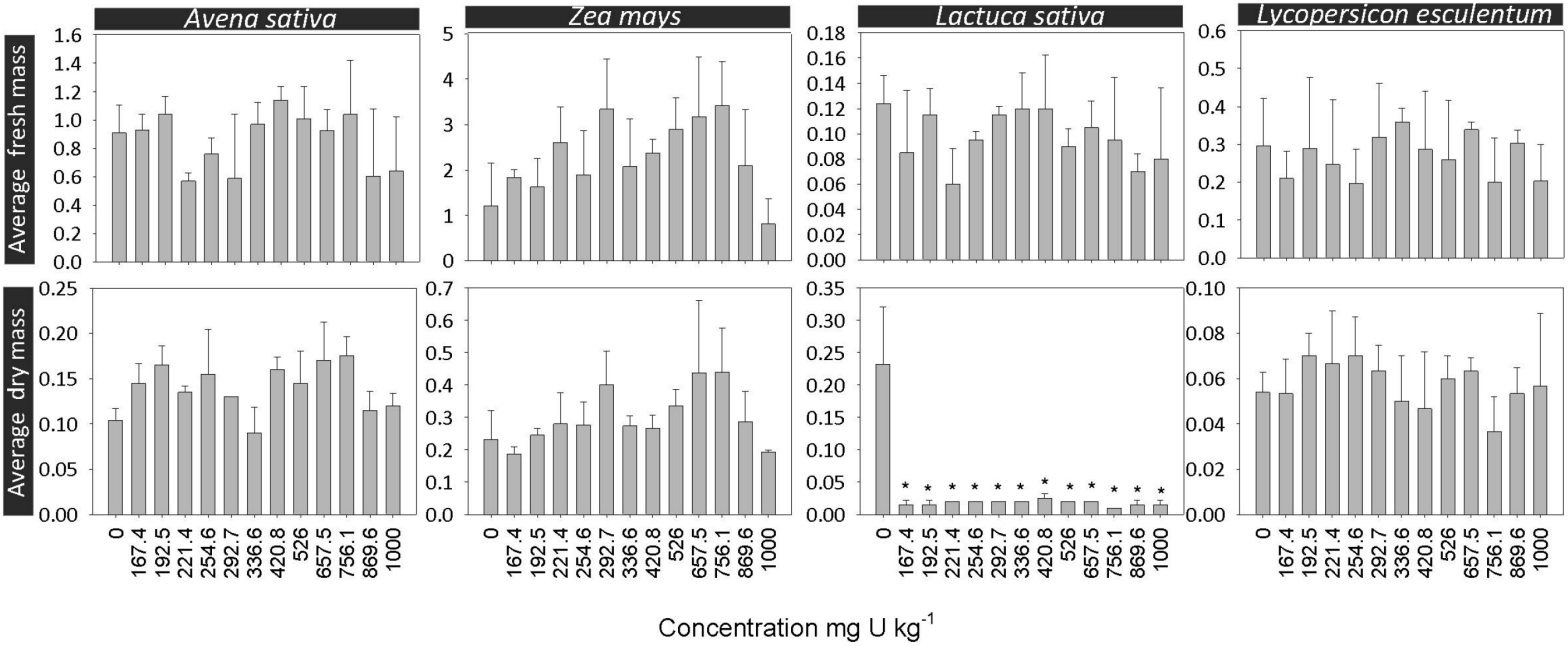
Growth of plants. Average values of fresh mass and dry mass in monocotyledonous, *Avena sativa and Zea mays* and in dicotyledonous species, *Lycopersicon esculentum and Lactuca sativa* grown in PTRS1 soil, contaminated with U. The error bars indicate the standard deviation. The asterisks point out significantly differences from the control (P<0.05).

With regard to production of fresh- and dry-mass, it was possible to perceive that the tested plants displayed different sensitivities to this metal. However, no significant differences were generally observed comparatively to the control, exception for *L. sativa* dry mass (H = 22.8, d.f. = 12, p = 0.029). Thus, and according to Figure 4, *L. sativa* was the most sensitive terrestrial plant to U. The high sensitivity of *L. sativa* was also found by Hubálek et al. [104] and Soudek [105]. This was probably caused by the high capacity of this species to bioaccumulate high concentrations of metals, including U [22].

The exposure of plants to metals, was already extensively studied, showing that these contaminants can induce biological effects on germination, growth and development, as well as, alterations in the nutrient profile of plants [22], [106]. However, only some studies (e.g., [66], [107] and others reviewed [98]) have assessed the ecotoxicological effects of U on terrestrial plant species.

Based on our study, once again was proved the diverse ecotoxicological outcomes for U effects on plant species, since no effects were observed, in the range of tested concentrations for the three evaluated endpoints (in three out of four species), in PTRS1 soil. Similar results were obtained by Sheppard and Sheppard [86] in acidic soils (Table 4), when testing the emergence and growth of wheatgrass *Elymus lanceolatus*. Like in our study, these authors did not observe any effect on this species (up to 1000 mg U kg soil_dw_^−1^). In opposition, Sheppard and Sheppard [81] revised data on U toxicity to terrestrial plants and reported EC_25_ values ranging from 300 to 500 mg U kg soil_dw_^−1^, considering only the most reliable studies. Stojanović et al. [108] also reported phytotoxic effects of U on *Zea mays* exposed, in different soil types, to 250, 500 and 1000 mg U kg soil_dw_^−1^, but especially at the highest concentration tested and in the most acidic soil. However, no statistical analysis of the data was performed in this study.

Soil properties are also the factors that most strongly affect U uptake and phytotoxic effects, [18], [109]–[111]. The bivalent uranyl ion (UO_2_^2+^) is sorbed to the negatively charged surfaces of clay minerals and organic compounds. In acidic soils subjected to pH increase, more negatively charged binding sites are available on mineral surfaces due to the progressive reduction of protons occupying these sites. However, pH values close to 6, like the one of PTRS1, favors U availability, since the concentrations of carbonates tends to increase, and U is released to the soil solution in the form of U-carbonate complexes [18]. The natural soil PTRS1, besides being acidic, has a lower clay content, which means lower binding sites for the bivalent uranyl ion (UO_2_^2+^), hence constraining U bioavailability. other soil properties and plant mechanisms may explain the reduced sensitivity of the plants in comparison with soil microbial parameters and invertebrates. Viehweger and Geipel [112] reported an increased U absorption by *Arabidopsis halleri* attributed to Fe deficiency in the medium of hydroponically grown plants. With respect to this metal, in the natural PTRS1 soil, the analyses done by Caetano et al. [49] showed that Fe surpassed the soil benchmark values proposed by two EPA regions (http://rais.ornl.gov/tools/eco_search.phphttp://rais.ornl.gov/tools/eco_search.php). In this sense, it is hypothesized that the high Fe content of the PTRS1 soil, may have also contributed for reducing the absorption of U by plants. As far as plant mechanisms are considered, in several studies reviewed by Mitchell et al. [113] the transport of U within plants was reduced and higher concentrations were consistently found in the roots. Using X-ray absorption spectroscopy (XAS) and transmission electron microscopy (TEM), Laurette et al. [114] observed that when plants are exposed to U and phosphates, needle-like U-phosphates are formed and precipitate, both outside and inside the cells, or persist in the subsurface of root tissues. The precipitation of U-phosphate complexes acts as a protective mechanism preventing U translocation to the shoots and leaves. This can also occur when the culture medium of the plants has no phosphate, since some plants are able to exudate phosphates. Further, U may be also absorbed like UO_2_^2+^ and linked to endogenous organophospate groups [114]. In opposition, when translocation occurs within plants, U has mainly formed U-carboxylated complexes. Plants can also exudate organic acids to the rhizosphere environment or UO_2_^2+^ may form complexes with endogenous compounds like malic, citric, oxalic and acetic acid [114]. In summary, the different resistance mechanisms described above could explain the lack of toxic effects observed for *A. sativa*, *Z. mays* and *L. esculentum*, in opposition to *L. sativa*. Most concerning is the fact that the majority of studies testing the phytotoxicity of U, including those performed by us, were made with the addition of nutrients solution, which increased the availability of phosphates to the test soil, likely decreasing the sensitivity of plants to U. Hence, to enhance the protection level of SSVs derived for plants, more assays with different plant species should be performed and the addition of nutrients should be prevented, or at least the tests may include replicates with and without nutrients.

## Derivation of a Soil Screening Value (SSV) for Uranium Applying Assessment Factors

The PNEC values obtained for U were based in EC_20_ and NOEC values varied between 15.5 and 23.3 mg kg soil_dw_^−1^, respectively (Table 5). These values were six to four times lower than the PNEC value suggested by Sheppard and Sheppard [86] (Table 5), which was 100 mg Ukg soil_dw_^−1^. In opposition, they are close to the lowest Canadian Soil Quality Guideline for both environment and human health (23 mg U kg soil_dw_^−1^). Thereby, while more ecotoxicological data is being obtained or other methods are being applied to derive soil screening values (SSVs) we prefer to be precautious by proposing a PNEC of 15.5 mg Kg^−1^ soil_dw_ as a SSV for U, in soils similar to the PTRS1. This SSV value is near the background value found in non-contaminated soils [8], [48], but not in some areas with naturally occurring U anomalies in soils, where concentrations ranging between 13–724 mg U kg soil_dw_^−1^ can be found [115].

**Table 5:** Soil quality guideline values derived for copper in Portugal, USA and Canada (mg U Kg^−1^ soildw).

Portugal				Canada	Other reference
Backgound concentrations	PNEC		Proposed SSV^b^	SQG_E_ c	
	NOEC	EC_20_			
7.8^a^	23.3	15.5	15.5	23	100^d^

a Caetano et al.[49];

b SSV - soil screening value;

c Canadian Soil Quality Guidelines for environmental health (SQGe), Scott-Fordsmand and Pedersen [116].;

d Sheppard and Sheppard [98].

## Conclusion

With the present study it was possible to generate a set of important ecotoxicological data for the derivation of a SSV for U, using a Portuguese natural soil representative of a granitic region, where this type of mine exploration occurred.

Soil enzyme activities were clearly inhibited by U. The obtained results depended not only on the concentrations of U but also on the properties of soil, which were likely responsible for the bioavailability of U and subsequent impairments on soil microbial population and, consequently, in their activity. Dehydrogenase and urease were particularly sensitive to U. Further, and comparatively to the remaining effect concentrations obtained/estimated for invertebrates and plants, the soil microbial parameters were more affected by U contamination^1^_._

The toxic effects of U in soil invertebrates were also confirmed, but the tested species showed a variable sensitivity to this metal. The increasing order of species sensitivity to U based on EC_50_ values for reproduction was *E. crypticus* > *E. andrei* > *F. candida*. However, if EC_20_ values are considered *F. candida* is the most sensitive invertebrate, since its EC_20_ value was 343.41 mg U kg soil_dw_^−1^, compared to 474.83 mg U kg soil_dw_^−1^ and 469.76 mg U kg soil_dw_^−1^ EC_20_ values estimated for *E. andrei* and *E. crypticus*, respectively. The EC_20_ values estimated were lower than the NOEC values for *E. andrei* and *F. candida*. Thus, the EC_20_ point estimate should be selected for the derivation of more protective SSVs. Relatively to the plants, the tested species showed no adverse effects caused by U in PTRS1, with the exception of *L. sativa* dry mass yield. Considering the results obtained, it was possible to verify a great variability between the EC_x_ values estimated in this study and those reported in the scientific literature. Multiple factors can contribute to this discordance, but probably at least for some species, soils physical and chemical properties were the main factors responsible for such differences. Although, this reinforces, at least in part, the importance of using natural soils representatives of the main types of soil from each region in ecotoxicological evaluations and for the derivation of SSVs, the data generated suggests that the SSV (15.5 mg Kg^−1^ soil_dw_) derived for U, was six times lower than the PNEC value proposed by other authors. Nevertheless, as mentioned previously, more data should be obtained following standard protocols.
