# Correction: Contribution for the Derivation of a Soil Screening Value (SSV) for Uranium, Using a Natural Reference Soil

**DOI:** 10.1371/journal.pone.0117749

**Published:** 2015-01-21

**Authors:** The PLOS ONE Staff

There are errors in the Author Contributions. The correct contributions are: Conceived and designed the experiments: ALC CRM RP FG EFS. Performed the experiments: ALC AG. Analyzed the data: ALC CRM RP. Contributed reagents/materials/analysis tools: FC FG EFS. Wrote the paper: ALC CRM RP FG EFS FC.
